# Respiratory virus is a real pathogen in immunocompetent community-acquired pneumonia: comparing to influenza like illness and volunteer controls

**DOI:** 10.1186/1471-2466-14-144

**Published:** 2014-09-02

**Authors:** Yangqing Zhan, Zifeng Yang, Rongchang Chen, Yutao Wang, Wenda Guan, Suishan Zhao

**Affiliations:** 1 Department of Respiratory Medicine, The First Affiliated Hospital of Guangzhou Medical University, State Key Laboratory of Respiratory Disease (Guangzhou Medical University, China), Guangzhou Institute of Respiratory disease, 151 Yanjiang Road, Guangzhou, PR, China; 2 Department of Clinical Virology, The First Affiliated Hospital of Guangzhou Medical University, State Key Laboratory of Respiratory Disease (Guangzhou Medical University, China), Guangzhou Institute of Respiratory disease, 151 Yanjiang Road, Guangzhou, PR, China

**Keywords:** Cell culture, Clinical feature, Community-acquired pneumonia, Seroconversion, Viral disease

## Abstract

**Background:**

Viral pathogens were more commonly reported than previously estimated in community-acquired pneumonia (CAP) patients. However, the real role of virus was still controversial.

**Methods:**

Consecutive adult patients with CAP between April and December, 2009 were prospectively enrolled. A four-fold or greater increase of IgG-titres against respiratory viruses in pair sera was tested by means of hemagglutination inhibition assay or indirect immunofluorescence. Swab samples were tested by cell culture and/or nucleic amplification tests. Viral etiology was considered definitive if at least one of the above tests was positive.

**Results:**

Viral etiology was established in fifty-two (34.9%) of 149 CAP patients, twenty-two (81.5%) of 27 influenza like illness patients, and none of 75 volunteer controls. Forty-seven CAP patients were infected by a single virus (24 influenza A virus, 5 influenza B, 10 parainfluenza virus type 3 [PIV-3], 2 PIV-1, 2 adenovirus, 2 human rhinovirus and 2 coronavirus OC43), five cases by two or three viruses co-infection. Fever ≥ 39°C (66.7%), fatigue (64.6%), and purulent sputum (52.1%) was the most common symptoms in viral pneumonia patients. On multivariate analysis, myalgia was included in the model for pneumonia associated with influenza infection. In the CURB-65 model only influenza infection was found independently associated with severe disease (CURB-65 score ≥ 3) out of variables, including age(years), sex, current smoking status, sick contact with febrile patients, numbers of comorbidity, presence of influenza infection, presence of PIV infection, with P = 0.021, OR 7.86 (95% CI 1.37-45.04).

**Conclusion:**

Respiratory virus was not a bystander, but pathogenic in pneumonia and was a common cause of CAP.

## Background

In China, pneumonia ranks fifth among all causes of death in humans. However, there are limited data regarding the etiology of community-acquired pneumonia (CAP) worldwide and in China, with about 17% to 48% unknown [1]. This may lead to inappropriate antimicrobial therapy and emergence of drug-resistant bacteria.

Since influenza virus was first isolated in ferrets from pneumonia patients in 1933 by Smith [2], viral etiology of pneumonia has attracted more and more attention. Recently, our ability to detect viral pathogens has dramatically improved after the introduction of highly sensitive nucleic amplification tests (NATs). Additionally, NATs has its superiority in detection of viruses that are difficult to grow in cell culture, such as human rhinovirus (HRV), human coronaviruses (HCoV), and new emerging pathogens human metapneumovirus (hMPV) and human bocavirus (HBoV).

Recently epidemiological surveys on etiology of CAP showed that respiratory viruses accounted for 15% to 56% of cases [3-5]. However, the real role of virus in pneumonia was few studied and still controversial [3,6]. It may partially due to poor sensitivity of most viral testing assays (except NATs). However, it was difficult to confirm the pathogenicity of virus tested by NATs. Thus, clinical features of specific viral pneumonia were not well described [4,5,7]. After combined the improvement in sensitivity and specificity of viral testing assay with more comprehensive design study, more valuable information will be available.

Moreover, because there is limited information concerning to the prevalence and clinical features of viral pneumonia, guideline of diagnosis and treatment of CAP does not provide much recommendation about the assessment and management of viral CAP.

In order to better understand the real role of respiratory virus in pneumonia and better manage the patients, we conducted a prospective observational study to reveal the viral etiology of adult CAP in Guangzhou, as compared with etiology of patients diagnosed with influenza like illness (ILI) and with volunteer controls.

## Methods

### Patients

Between April and December, 2009, consecutive adult patients admitted to the First Affiliated Hospital of Guangzhou Medical University and diagnosed with CAP within 14 days from onset were studied. They were sampled for throat swabs at enrollment and paired sera by at least two weeks interval. CAP was defined as the presence of a new infiltrate on the chest radiographs, together with a new cough or sputum or change in respiratory symptoms, or fever, or sign of consolidation of lung or rales, or leukocytosis (>10 × 10^9^/L) or leucopenia (<4 × 10^9^/L) [8]. No alternative diagnosis was responsible to the new infiltrate during follow-up. Exclusion criteria was: 1) immunosuppression (e.g. human immunodeficiency virus infection); 2) previous organ transplantation; 3) immunosuppressive therapy, defined as daily doses 20 mg prednisolone or equivalent for 2 weeks; 4) any dose of an immunosuppressive combination regimen, including azathioprine, cyclosporin and/or cyclophosphamide; 5) treating cancer; 6) lung abscess, aspiration pneumonia and tuberculosis. Pregnant women, patients who were released from hospital within 14 days and who didn’t signature the consent were excluded.

Additionally, ILI patients were enrolled. It was defined as an acute illness within 14 days, with fever (≥38°C), two constitutional symptoms (chills, headache, myalgia or fatigue) and one respiratory symptom (cough, sore throat or coryza) [9], without evidence of pneumonia. Throat swab samples were taken at enrollment and paired sera were taken by two weeks interval.

Both pneumonia patients and ILI patients were followed up via telephone or interview for up to 30 days. All data were recorded by a trained doctor, who was blinded to the results of viral detection.

Moreover, volunteer controls without clues of acute illnesses within one month were also enrolled and sampled for throat swabs.

The study was approved by the ethics committee of The First Affiliated Hospital of Guangzhou Medical University and informed consent was obtained for all subjects.

### Viral testing

Paired sera were routinely performed by hemagglutination inhibition assay [10] for detection of seasonal influenza virus type A and B (Flu A and B) and pandemic (H1N1) 2009 influenza A virus (A[H1N1]pdm09), indirect immunofluorescence (EUROIMMUN, Lübeck, German) for detection of parainfluenza virus type 1, 2, 3 and 4 (PIV-1,2,3,4), adenovirus (Adv) and respiratory syncytial virus (RSV). Four-fold or greater increase of IgG-titres was defined as positive. Swab samples were processed for study of viruses mentioned above through isolation of viruses in shell-vial cell culture system (PIV-4 is excluded) and for detection of nucleic acids by reverse transcription polymerase chain reaction (RT-PCR) assays (PIV-2 and PIV-4 is excluded) [11-13]. Swab samples were also tested for HRV 1/2/3/4, HCoV (229E, OC43, NL63, HKU1), hMPV and HBoV by Taqman real-time RT-PCR (rRT-PCR) [14], in accordance with the manufacturer’s protocol (Guangzhou HuYanSuo Medical Technology Co., Ltd, China). Viral etiology was considered definitive if at least one of the above tests was positive.

### Statistical analysis

Statistical software (SPSS 13.0; SPSS, Chicago, IL, USA) was employed for statistical analysis. Quantitative data were presented as median and interquartile range (IQR) and compared by non-parametric Kruskal-Wallis test. The categorical variables were reported as frequencies and percentages and compared using the Fisher’s exact or Chi-square test.

Logistic regression analysis was applied to test whether or not certain clinical features on admission were associated with specific virus infection. The presence or absence of specific virus infection was analysed. The other variables included age (years), sex, current smoking status, sick contact with febrile patients, numbers of comorbidity, presence of fever ≥ 39°C, myalgia, headache, fatigue, dry cough, coryza, sore throat, hemoptysis, chest tightness or pain, dyspnea, and the presence of neutrophilia (defined as >8 × 10^9^/L), leukocytosis (defined as >10 × 10^9^/L) and lymphopenia (defined as <0.8 × 10^9^/L).

Logistic regression analysis was also applied to test whether demographic features or specific virus infection were associated with severe disease, which was defined as CURB-65 score equal or greater than 3 at admission. There are five risk factors in CURB-65, including confusion of new onset, blood urea nitrogen greater than 7 mmol/l, respiratory rate of 30 breaths per minute or greater, systolic blood pressure less than 90 mmHg or diastolic blood pressure less than 60 mmHg, age 65 or older. Each risk factor scores one point, for a maximum score of 5.The other variables included in the model were: age (years), sex, current smoking status, sick contact with febrile patients, numbers of comorbidity, presence of influenza infection, presence of PIV infection.

For all logistic regression analysis, variables with a P value < 0.1 in binary analysis were entered in the multivariate analysis. The level of significance was set at <0.05.

## Results

Overall, 261 individuals were enrolled, including 159 cases of CAP, 27 cases of ILI and 75 cases of volunteer. However, 10 cases of CAP were excluded (2 obstructive pneumonia, 3 pulmonary tuberculosis, 4 immunosuppressed patients and 1 sheep brucellosis). Eighty-nine patients had at least one underlying condition (Table 1). Swab samples were available in all patients and volunteers, paired sera in 70 cases of CAP patients and in all ILI patients.

**Table 1:** Demographic data and comorbidity of patients and volunteer controls

	**CAP**	**ILI**	**Volunteers**	**P values**^**†**^
Cases	149	27	75	
Age (median [IQR] yrs)	60(35–77)	31(25–34)	53(45–62)	0.829
Male/female	84/65	16/11	40/35	0.666
Comorbidity*	89(59.7)	6(22.2)	40(53.3)	0.360
COPD	38(25.5)	1(3.7)	15(20.0)	0.360
Asthma	6(4.0)	1(3.7)	6(8.0)	0.212
Bronchiectasis	8(5.4)	0	4(5.3)	0.991
Hypertention	37(24.8)	0	6(8.0)	0.003
Heart cerebro- vascular diseases	28(18.8)	1(3.7)	13(17.3)	0.790
Diabete mellitus	14(9.4)	0	6(8.0)	0.730
Hepatic disease	5(3.4)	1(3.7)	3(4.0)	0.892
Renal disease	2(1.3)	0	1(1.3)	0.542
Other diseases	21(14.1)	2(7.4)	6(8.0)	0.186

Note: *All data were showed as numbers (%) unless otherwise specified. ^†^Comparisons were made between groups CAP and volunteers by non-parametric Kruskal-Wallis test for quantitative characteristics and Fisher’s exact or Chi-square test for categorical variables, respectively.

**Figure 1:**
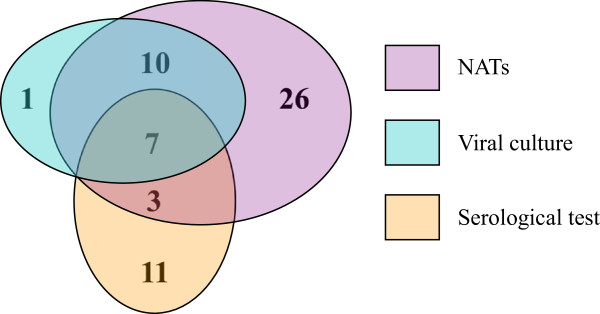
**Number of viruses in community-acquired pneumonia patients detected by three different assays respectively.** (NATs: Nucleic amplification tests).

Viral etiology was established in 52 (34.9%) of 149 CAP patients, in 22 (81.5%) of 27 ILI patients, as shown in Table 2. All volunteers were virus negative. Among 58 viruses from CAP patients, 18 viruses were detected by shell-vial cell culture system, 47 viruses by NATs and 20 viruses by serological survey, respectively (Figure 1).

**Table 2:** Etiology of community acquired pneumonia and influenza like illness

**Pathogens**	**Cases**	**Percentage (%)**
CAP patients	149	
Pure virus infection	49	30.2
Flu A	23	15.4
Flu B	5	3.4
ADV	2	1.3
PIV-1	2	1.3
PIV-3	9	6.0
HCoV OC43	2	1.3
HRV	2	1.3
Flu A + PIV-1	1	0.7
Flu B + PIV-3	1	0.7
PIV-3+ HCoV OC43	1	0.7
Flu A + PIV-3 + HCoV OC43	1	0.7
Virus and bacteria co-infection	3	2.0
Flu A + Escherichia coli	1	0.7
Flu A + ADV + Escherichia coli	1	0.7
PIV-3+ Haemophilus parainfluenzae	1	0.7
Pure bacteria infection	3	2.0
Haemophilus influenzae	1	0.7
Haemophilus hemolyticus	1	0.7
Stenotrophomonas maltophilia	1	0.7
ILI patients	27	
Flu A	14	51.9
Flu B	4	18.5
PIV3	1	3.7
Flu A + Flu B	2	7.4
Flu A + PIV1	1	3.7
Flu A + PIV3	1	3.7

Sputum bacterial culture was performed for the clinical need in 77 hospitalized patients with CAP and results was also recorded. Six patients yield a positive result (Table 2). Three out of six patients were virus positive.

Clinical features and severity of illness in CAP patients were summarized in Table 3 and Table 4, respectively. Some Flu A or PIV-3 infected patients manifested hemoptysis and chest pain. Dyspnea and gastrointestinal symptoms were also common in Flu and PIV-3 positive CAP patients. Compared with virus negative patients, sorethroat and fatigue was more common, leukocytosis and neutrophilia was less common in viral pneumonia patients, although fatigue and neutrophilia had no statistical significance. It seemed that length of hospital stay was longer, intensive care requirement and 30-day mortality was higher in virus positive patients, however, there was no statistical significance. Table 5 showed the significant features associated with viral pneumonia or severe disease on multivariate analysis. On viral pneumonia model, leukocytosis was negative correlated with any virus infection. While, myalgia was included in the model for pneumonia associated with influenza infection. In the CURB-65 model, only influenza infection was found independently associated with severe disease (CURB-65 score ≥ 3), with P = 0.021, Odds Ratio (OR) 7.86 (95% Confidence interval [CI] 1.37-45.04).

**Table 3:** Clinical characteristics of community acquired pneumonia patients with different etiology

	**Flu A**	**Flu B**	**ADV**	**PIV-1**	**PIV-3**	**Rhv**	**HCoV OC43**	**Dual-virus**	**Virus positive**	**Virus negative**	P values**
Cases	24	5	2	2	10	2	2	5	52	97	
Demographic data											
Age (Median [IQR], yrs)	74(32–80)	47(36–73)	21(20–23)	72(65–78)	63(40–70)	72(66–77)	71(67–74)	59(45–65)	62(32–79)	60(36–75)	0.961
Male/Female	15/9	3/2	1/1	2/0	3/7	2/0	0/2	3/1	29/23	55/42	0.913
Sick contact*	5(20.8)	1(20.0)	0	0	1(10.0)	0	0	2(40.0)	9(17.3)	10(10.3)	0.222
Current smoking	3(12.5)	1(20.0)	0	0	2(20.0)	0	0	1(25.0)	7(13.5)	19(19.6)	0.348
Clinical symptoms											
Fever ≥ 39°C	14(58.3)	3(60.0)	2(100)	1(50.0)	6(60.0)	1(50.0)	0	4(80.0)	31(59.6)	49(50.5)	0.288
Headache	8(33.3)	3(60.0)	1(50.0)	0	9(90.0)	1(50.0)	0	3(60.0)	25(48.1)	47(48.5)	0.965
Mylgia	10(41.7)	3(60.0)	1(50.0)	0	0	0	0	3(60.0)	17(32.7)	33(34.0)	0.870
Fatigue	14(58.3)	4(80.0)	1(50.0)	1(50.0)	8(80.0)	1(50.0)	0	3(60.0)	32(61.5)	46(47.4)	0.100
Coryza	10(41.7)	2(40.0)	1(50.0)	0	4(40.0)	1(50.0)	0	3(60.0)	21(40.4)	28(28.9)	0.154
Sore throat	10(41.7)	2(40.0)	2(100.0)	0	4(40.0)	2(100)	1(50.0)	4(80.0)	25(48.1)	30(30.9)	0.038
Dry cough	2(8.3)	1(20.0)	1(50.0)	0	0	0	0	0	4(7.7)	9(9.3)	0.982
Purulent sputum	12(50.0)	2(40.0)	1(50.0)	1(50.0)	5(50.0)	1(50.0)	0	4(80.0)	26(50.0)	49(50.5)	0.777
Hemoptysis	2(8.3)	0	0	0	2(20.0)	0	0	0	4(7.7)	3(3.1)	0.391
Chest tightness or pain	6(25.0)	0	0	0	1(10.0)	2(100)	1(50.0)	0	10(19.2)	22(22.7)	0.625
Dyspnea	12(50.0)	3(60.0)	0	1(50.0)	4(40.0)	0	1(50.0)	0	21(40.4)	33(34.0)	0.441
Gastrointestinal symptoms	5(20.8)	1(20.0)	0	0	1(10.0)	1(50.0)	0	1(20)	9(17.3)	15(15.5)	0.770
Laboratory results											
Leukocytosis^†^	8(33.3)	0	1(50.0)	1(50.0)	1(10.0)	1(50.0)	1(50.0)	0	13(25.0)	43(44.8)	0.020
Neutrophilia^§^	8(33.3)	0	1(50.0)	1(50.0)	1(10.0)	1(50.0)	1(50.0)	0	13(25.0)	39(40.6)	0.063
Lymphopenia^‡^	6(25.0)	0	0	1(50.0)	1(10.0)	0	0	0	7(13.5)	9(9.4)	0.432
ESR > 20 mm/h^||^	9(81.8)	NA	0	1(100.0)	4(80.0)	1(100)	1(100)	2(66.6)	18(78.3)	45(83.3)	0.597
PaO2 < 80 mmHg^||^	10(41.7)	2(40.0)	1(50.0)	1(50.0)	3(75.0)	2(100)	1(50.0)	1(50.0)	21(67.7)	26(61.9)	0.607

Note: *All data were showed as numbers (%) unless otherwise specified. ^†^Leukocytosis was defined as leukocyte count >10 × 10^9^/L. ^§^Neutrophilia was defined as neutrophil count >8 × 10^9^/L. ^‡^Lymphopenia was defined as lymphocyte count <0.8 × 10^9^/L. ^||^Data were available only in some patients. **Comparisons were made between virus positive patients and virus negative patients by non-parametric Kruskal-Wallis test for quantitative characteristics and Fisher’s exact or Chi-square test for categorical variables, respectively. NA: Data is not available. ESR: erythrocyte sedimentation rate. Gastrointestinal symptoms included nausea, vomiting, diarrhea, abdominal pain, and abdominal distention.

**Table 4:** Severity of illness in community acquired pneumonia patients with different etiology

	**Flu A**	**Flu B**	**ADV**	**PIV-1**	**PIV-3**	**Rhv**	**HCoV OC43**	**Dual-virus**	**Virus positive**	**Virus negative**	**P values**^**†**^
Cases	24	5	2	2	10	2	2	5	52	97	
Hospitalization^§^*	21(87.5)	4(80.0)	1(50.0)	2(100)	8(80.0)	2(100)	2(100)	4(80.0)	42(80.8)	76(78.4)	0.729
Length of hospital stay (Median [IQR], days)	11(9–18)	13.5(9–17)	21	12(9.5-14.5)	10.5(6–12.8)	11(13–15)	19(18.5-19.5)	10(8.5-12.8)	11(8.8-17.3)	10(7.8-15)	0.268
Ventilation	2(8.3)	1(20.0)	0	0	0	0	0	0	3(5.8)	3(3.1)	0.722
Intensive care requirement	2(8.3)	1(20.0)	0	0	1(10.0)	0	0	0	4(7.7)	5(5.2)	0.720
ICU admission	1(4.2)	0	0	0	0	0	0	0	1(1.9)	3(3.1)	0.912
30-days mortality	1(4.2)	1(20.0)	0	0	1(10.0)	0	0	0	3(5.7)	2(2.1)	0.343

Note: *All data were showed as numbers (%) unless otherwise specified. ^†^Comparisons were made between virus positive patients and virus negative patients by non-parametric Kruskal-Wallis test for quantitative characteristics and Fisher’s exact or Chi-square test for categorical variables, respectively. ^§^Some patients denied hospitalization and were treated in Outpatient Clinic or Emergency Department. ICU: intensive care unit.

**Table 5:** Significant features associated with viral pneumonia or severe disease (CURB-65 score equal or greater than 3) on multivariate analysis

**Etiology**	**Clinical features**	**OR (95% CI)**	**P value**
Viral pneumonia model			
Any virus infection	Leukocytosis	0.40 (0.19 to 0.85)	0.016
Influenza infection	Myalgia	2.27 (1.03 to 5.01)	0.042
PIV infection	Myalgia	0.14 (0.03 to 0.67)	0.014
	Fatigue	4.38 (1.25 to 15.23)	0.020
	Leukocytosis	0.18 (0.04 to 0.86)	0.032
CURB-65 model	Influenza infection	7.86 (1.37 to 45.04)	0.021

Note: Leukocytosis was defined as leukocyte count >10 × 10^9^/L.

All CAP patients received antibiotics. However, few patients were prescribed for antiviral agents such as oseltamivir or zanamivir by clinician. Seven patients (two of which was virus positive) received short course of intravenous ribavirin before their admission to our hospital. Only one A(H1N1)pdm09 infected patient received oral oseltamivir before and during his hospitalization in our hospital. Finally, all but five CAP patients recovered during our one month observation. Clinical course of four CAP patients (one was Flu B positive, one was PIV-3 positive, none received antiviral drugs) went deteriorated. They denied going on hospital stay and died within 30 days after hospitalization. One COPD patient who was diagnosed with seasonal influenza A pneumonia, developed respiratory failure and denied invasive ventilation and ICU admission. Ultimately, he died.

## Discussion

Three methodological aspects, including full sets of viral tests applied, immunocompetent patients enrolled, and two control groups, made our study different from previous published data [3-5,15-17]. Base on our methodological design, finally, we found that virus detected in pneumonia was not a bystander, but pathogenic. Respiratory virus accounted for about one-third of pathogens in CAP. Hence, it was a common cause of CAP. In view of high prevalence of viral CAP, there was an urgent need to consider routine laboratory detection in hospitalized CAP patients for an adequate diagnosis of respiratory viruses. In addition, considering positive correlation between influenza infection and severe disease, we recommended testing for influenza virus routinely in severe individuals, especially during the active period of influenza activity.

Virus in pneumonia may be a bystander, but not pathogenic. In Johnstone’s and de Roux’s reports, though aetiology of nonimmunocompromised CAP patients were reported, the real role of virus was not determined [5,16].Whether samples obtained from upper respiratory tract can really mirror the true situation occurred in the lower respiratory tract and lung, it needs to be further studied. Hence, two control groups were introduced in the present study to answer this question. One of the purposes of enrolment of these two control groups was to describe inapparent infection in volunteer individuals without clues of acute respiratory illness within one month and a higher rate of viral infection in ILI patients. Another question was that the extreme sensitivity of the NATs was thought to be due to false-positive previously. Then, volunteer controls in our study turned to be virus negative, just what we expected. Comparison of positive rate of virus between two control groups and CAP patients in our study revealed that false positive of NATs did not appeared in our study. Second, in both ILI and pneumonia patients, seroconversion of IgG did not happen in all viral positive patients. Similarly, in report by Gencay, seroconversion of IgG was found only in 41% of patients diagnosed with lower respiratory tract viral infection [18]. Hence, even if four-fold elevation of virus specific antibody titer did not appear, the positive results from NATs were deemed to be the pathogen of current pneumonia. Similar to Jennings’s finding [3], positive results of virus by NATs in CAP should be considered as the pathogens.

In our study, viral CAP was more common than other reports worldwide [3-5,7,15,16,19-21]. We speculated that it may directly manifest the real incidence of viral infection in CAP locally in Guangzhou, southern of China, based on our methodological aspects mentioned above. However, there were 12 A(H1N1)pdm09, and influenza virus accounted for two-thirds of viral pathogens in CAP in our study. Hence, higher prevalence of viral CAP might be partially influenced by A(H1N1)pdm09 pandemic [22]. In addition, comprehensive viral testing methods and viral pathogens improved yields of virus and also contributed to a higher proportion of viral pneumonia.

In our cohort, Flu A and PIV-3 was the most common virus of CAP. Viral pattern was similar to the local influenza activity [23]. Similarly, influenza virus was shown to be predominant viral pathogen in CAP in Spain [5,16], in patients with acute exacerbations of COPD and concomitant pneumonia in Hongkong [24], while in New Zealand, rhinovirus was the most commonly identified pathogen, followed by influenza virus and RSV [3]. Difference in the prevalence of specific virus infection among studies may relate to diverse geographical, climate, activity of the virus pandemic in the community locally, testing assays applied and the viral pattern studied, etc.

Likewise, clinical features varied among studies. Jennings in New Zealand reported that the presence of myalgia was associated with pneumonia caused by any respiratory virus and influenza pneumonia [3]. The presence of chest pain and leukocytosis had been found to be far less common in those patients with a viral infection than in those with a bacterial infection [4,5]. It was similar in our study that leukocytosis was less common in virus infected patients, when compared to viral negative patients. While, Roux demonstrated that chronic heart failure and the absence of expectoration was associated with pure viral pneumonia when comparing to pneumococcal CAP [16]. However, although several variables were associated with some types of pathogen, clinical characteristics were unable to reliably distinguish viral pneumonia from viral negative pneumonia or bacteria pneumonia. All of these differences were clinically insignificant unless combined with the detection of viruses. It should be noted that all of the differences identified in the clinical characteristics among studies, may also be related to diverse geographical, cultural, and healthcare environments, though, this has not been confirmed [25].

Majority of viral pneumonia patients in our study recovered from illness without the aid of antiviral agents, which was similar to the clinical course of upper respiratory tract viral infections. Viral pneumonia seemed to be a self-limited illness. However, one influenza pneumonia patients in our study died, because of worsen of comorbidity. Also, clinical course of other two patients with viral pneumonia went deteriorated and died after discharge from hospital. However, illness in virus positive patients was similar to virus negative patients in length of hospital stay, intensive care requirement and 30-day mortality. Hence, one of our puzzled questions remained that among viral pneumonia patients, who needed to be treated with antiviral agents? Until now, only influenza can be effectively treated with neuraminidase inhibitors. Obviously, considering the high prevalence of viral CAP in Guangzhou and influenza infection as an independent variable in the severe disease model, routine laboratory detection should be taken in hospitalized CAP patients at admission for an adequate diagnosis of respiratory viruses, especially influenza virus in severe individuals. Then they may be benefited from antiviral treatment, if the latter was conducted in early stage [26].

Another question was that did all patients with viral community-acquired pneumonia, especially those without evidence of bacteria infection, need to be treated with antibiotics? Plenty of evidences had shown that virus infection predisposes the respiratory tract to superinfection by another pathogen, with bacteria the most common [27-29]. However, they were not benefited from preventive prescription of antibiotics [30]. Antibiotics in these patients had been reported to lead to the occurrence of antibiotic resistance in clinically relevant bacteria [31]. In our study, three pneumonia patients had virus and bacteria co-infection. They were not treated with any antiviral drug. With the “aid” of antibiotics, they recovered from illness. There was not enough information to illustrate the real role of antibiotics in the treatment of viral pneumonia in our study yet. Hence, further randomised placebo-controlled trials of antibiotic treatment for adult viral pneumonia, similar to that in the paediatrics [32], is needed to help answer the question.

Still, there were several limitations in this study. First, only swab sample was tested for viral culture and NATs. Increasing the sample types, such as nasopharyngeal aspirates, bronchoalveolar lavage fluid and sputum, may improve the yield of virus. Although multiple methods were applied, we were undoubtedly still underestimating the prevalence of respiratory virus infection and other types of pathogens. Hence, viral negative patients could not be ruled out from other viruses’ infection, which was outside the scope of testing in our study. Second, bacteria and atypical pathogens were not studied. Hence, comparisons of clinical characteristics were only made between virus positive patients and virus negative patients. Finally, only three seasons were covered in our study. A longer time that lasted two or more years and more patients enrolled may provide us more useful information. However, further work is ongoing to describe the epidemiological and clinical characteristics of viral CAP deeply.

## Conclusion

Respiratory virus was not a bystander, but pathogenic in pneumonia. It was a common cause of CAP. We recommended testing for influenza virus routinely in severe individuals, especially during the active period of influenza activity.

## Abbreviations

CAP: Community-acquired pneumonia; NATs: Nucleic amplification tests; HCoV: Human coronavirus; HRV: Human Rhinovirus; hMPV: Human metapneumovirus; HBoV: Human bocavirus; ILI: Influenza like illness; Flu A: Influenza virus type A; A(H1N1)pdm09: Pandemic (H1N1) 2009 influenza A virus; PIV: Parainfluenza virus; Adv: Adenovirus; RSV: Respiratory syncytial virus; RT-PCR: Reverse transcription polymerase chain reaction; rRT-PCR: Real-time reverse transcription polymerase chain reaction; IQR: Interquartile range; OR: Odds ratio; CI: Confidence interval.

## Competing interests

The authors declare that they have no competing interests.

## Authors’ contributions

Drs, YQ Zhan, ZF Yang, RC Chen were responsible for clinical management of the patient, collection and interpretation of clinical data, Drs YT Wang, WD Guan, SS Zhao were participated in the interpretation of virology data. All authors participated in the discussion of article and contributed to the drafting of the manuscript. All authors read and approved the final manuscript.

## Pre-publication history

The pre-publication history for this paper can be accessed here:

http://www.biomedcentral.com/1471-2466/14/144/prepub
